# Screening circular RNA expression patterns following focal cerebral ischemia in mice

**DOI:** 10.18632/oncotarget.21238

**Published:** 2017-09-23

**Authors:** Cuiying Liu, Chencheng Zhang, Jian Yang, Xiaokun Geng, Huishan Du, Xunming Ji, Heng Zhao

**Affiliations:** ^1^ China-America Institute of Neuroscience, Beijing Luhe Hospital, Capital Medical University, Beijing, China; ^2^ Department of Neurology, Beijing Luhe Hospital, Capital Medical University, Beijing, China; ^3^ Department of Neurosurgery, Xuanwu Hospital, Capital Medical University, Beijing, China; ^4^ Department of Neurosurgery, Stanford University, Stanford, CA, USA

**Keywords:** stroke, middle cerebral artery occlusion, circular RNA, microRNA, microarray

## Abstract

Circular RNAs (circRNAs) have been demonstrated to act as microRNA (miRNA) sponges and they play important roles in regulating gene expression through a circRNA-miRNA-gene pathway. The specific roles of circRNAs in the pathogenesis of cerebral ischemia, however, are still unclear. Thus, the aim of this study is to determine circRNA expression profiles in the ischemic brain after stroke, which was induced by 45 min of transient middle cerebral artery occlusion (MCAO). The results from the circRNA microarrays revealed that 1027 circRNAs were significantly altered 48 hours after reperfusion in the ischemic brain compared with the sham group. Among them, 914 circRNAs were significantly upregulated, and the remaining 113 were significantly downregulated. In addition, the expressions of the three selected circRNAs, mmu_circRNA_40001, mmu_circRNA_013120, and mmu_circRNA_40806, were verified using quantitative real-time polymerase chain reaction (qRT-PCR). After predicting their target genes, the Kyoto Encyclopedia of Genes and Genomes (KEGG) and Gene Ontology (GO) analyses were further used to predict the associated significant cell signaling pathways and functions. The results show that the most enriched pathways are associated with the Rap1 signaling pathway and the Hippo signaling pathway, which regulate cell survival and death. Finally, we constructed an interaction network of circRNA-miRNA-target genes, including 13 miRNAs and their corresponding genes, indicating that changes in circRNA are associated with genes related with brain injury and recovery. In conclusion, circRNAs are complicated in the pathological development of brain injury after stroke, suggesting novel diagnostic and therapeutic targets for stroke therapy.

## INTRODUCTION

Stroke is one of the leading causes of death and disability worldwide [1]. Currently, the FDA-approved thrombolytic therapy of recombinant tissue plasminogen activator (rt-PA) is available for only a small percentage of stroke patients, due to its narrow therapeutic time window of 4.5 hours after stroke onset [2]. Thus, it is urgent to understand the pathogenesis and underlying mechanisms of stroke-induced brain injury to develop novel diagnostic and therapeutic targets for stroke patients. Unlike the well-known linear RNA, circular RNAs (circRNAs) are single-stranded RNA molecules that form circles through covalent bonding [3]. This structure is a closed ring with neither a 5’cap nor 3’tail, which may prevent it from being degraded by RNA exonuclease, allowing it to maintain stable expression [4, 5]. CircRNAs mainly derive from the exonic regions of protein-coding genes and are stable with variety of length. Endogenous circRNAs were recently discovered to act as miRNA sponges, which binded to miRNAs and consequently repressed their function [6]. In addition, circRNAs also regulate their parental gene expressions. Accumulating evidence has demonstrated that circRNAs are involved in tissue and disease developments, including neuronal development, Alzheimer’s Disease, cardiovascular diseases, and cancers [7–13]. However, the research articles regarding to circRNA in stroke have not been reported.

The purpose of this current study is to reveal whether circRNAs, as a new class of non-coding RNA, are implicated in brain injury after stroke. We aim to predict their potential roles in ischemic brain pathologies by analyzing their expression changes after stroke, and their relationships with miRNA and mRNA. As previous studies have shown that circRNAs are largely expressed in brain tissues, and they are involved in brain disorders and development, as well as in cardiovascular diseases that are associated with endothelial functions, we assume that circRNAs must play important roles that require to be revealed. Therefore, we will identify differentially expressed circRNAs in ischemic brains and predicted their functions by constructing circRNA-miRNA-gene networks according to available bioinformatics. We wish that we could provide insights from current animal models for future clinical studies by identification of circRNA as potential biomarkers and diagnostic tools for stroke patients.

## RESULTS

### Expression pattern of circRNA in the ischemic brain after stroke

After 45min ischemia with 48h reperfusion, the behavioral test of mice is measured (data no shown). Furthermore, we also observed whether there was obvious edema in the ischemic ipsilateral brain. Only ischemic core of the brain tissue with high behavioral test and obvious edema were dissected for next RNA extraction and microarray assays.

**Figure 1:**
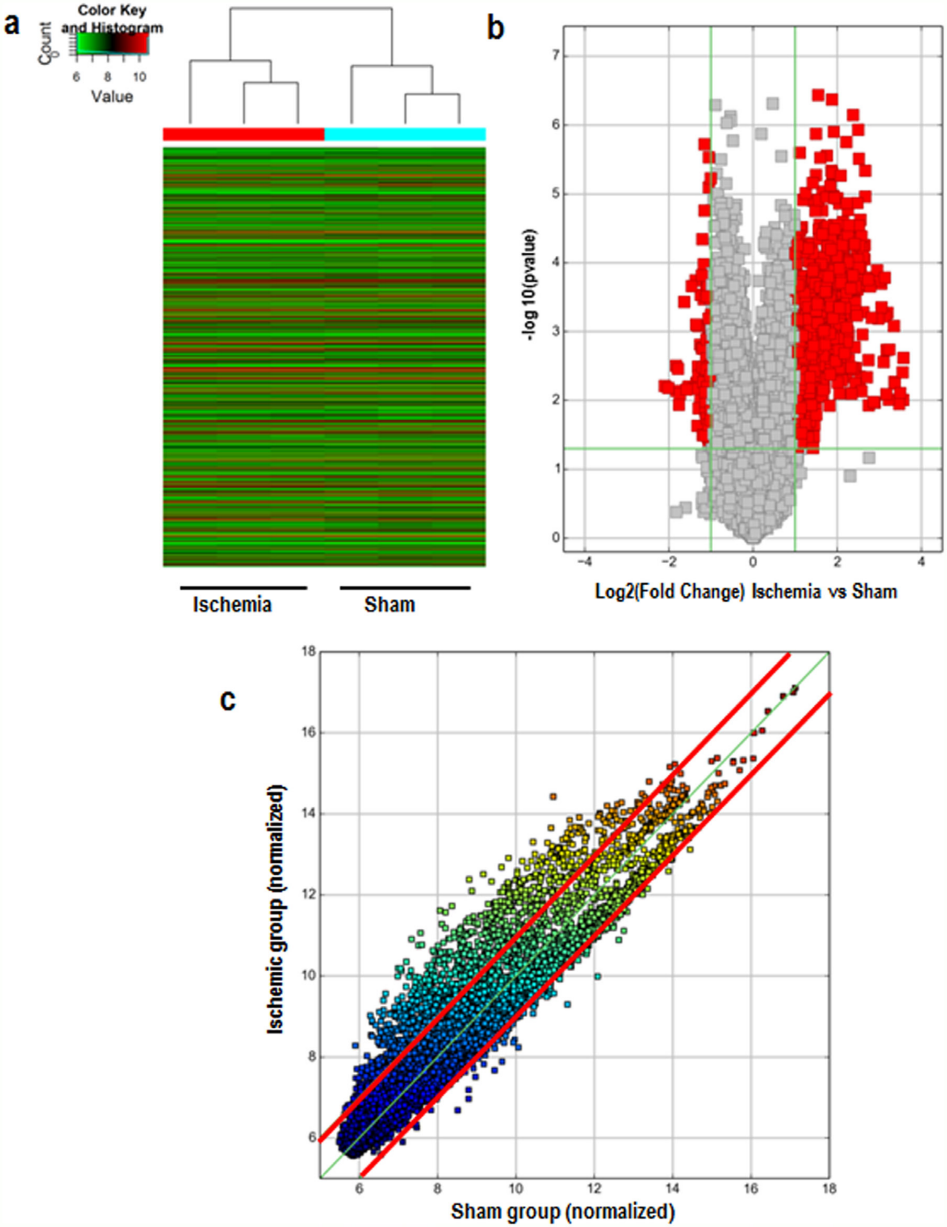
Differences in circRNA expression profiles between the ischemic and sham groups **(a)** Hierarchical cluster analysis of all differentially expressed circRNAs. Each column represents a sample, and each row represents a circRNA. Red strips represent high relative expressions and green strips represent low relative expressions, n=3 per group. **(b)** Volcano plots show the distributions of circRNAs between the ischemic and sham groups. The horizontal line corresponds to a 2-fold (log2 scaled) change up or down, and the vertical line represents a P-value of less than 0.05 (− log10 scaled). The red points on the plot represent the differentially expressed circRNAs with a 2-fold change up or down in expressed circRNAs with statistical significance (P < 0.05). **(c)** The scatter plot shows the differences in circRNA expression between the ischemic and sham groups. The values plotted on the X and Y axes are the averaged normalized signal values between the ischemic and sham groups (log2 scaled). CircRNAs in the scatter plot above the top red line and below the bottom red line indicate a greater than 2 fold change of circRNAs between the two compared samples.

**Figure 2:**
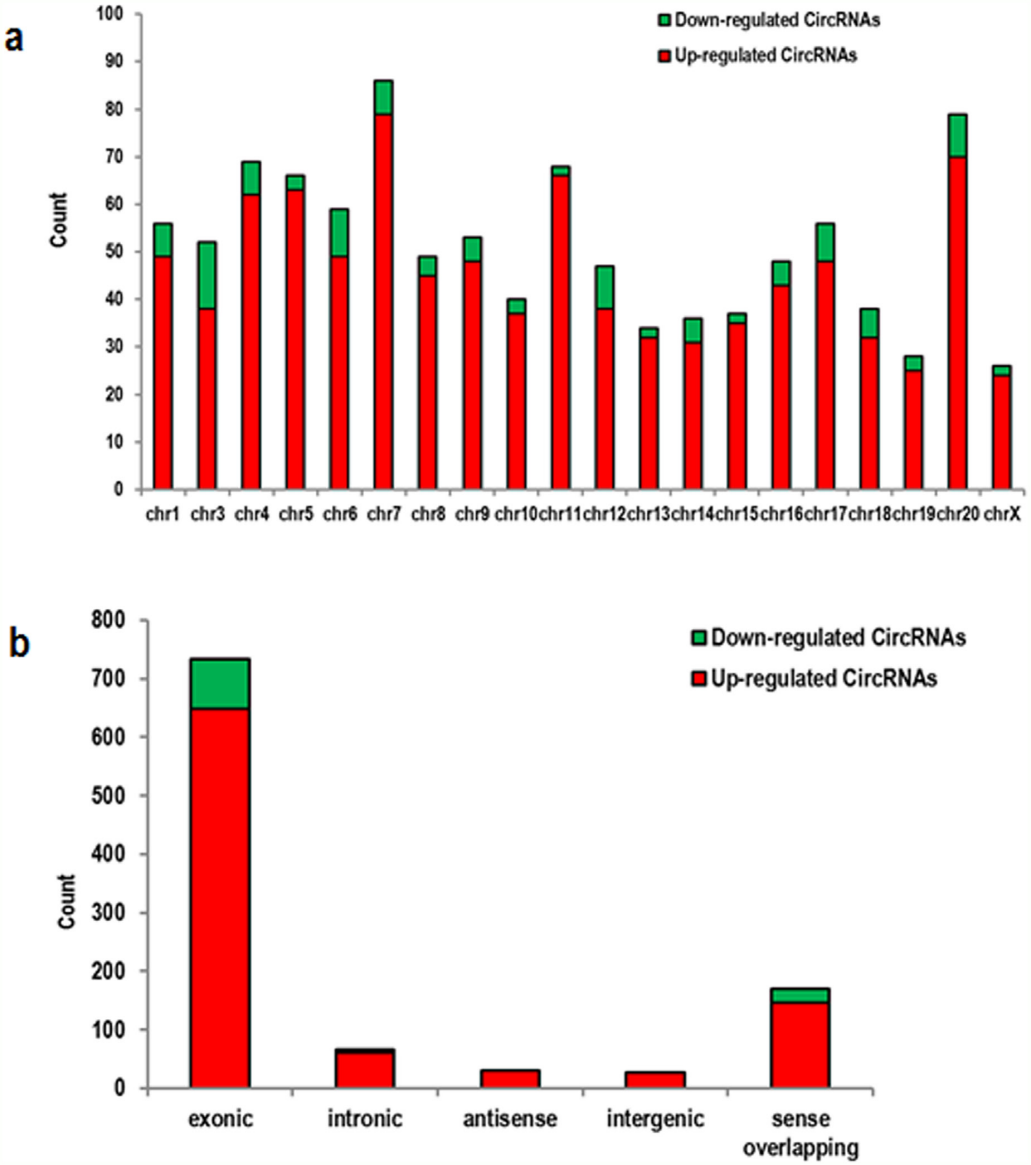
Characterizations in circRNA expression profiles between the ischemic and sham groups **(a)** The bar diagram shows the circRNA expressions on different chromosomes. **(b)** The distribution of differentially expressed circRNAs on different regions of the mouse chromosomes after stroke, including in exon, intron, intergenic region, sense, and antisense DNA. Most of the differentially expressed circRNAs originated from the exons.

Arraystar Mouse circRNA arrays were carried out in order to profile circRNA expression in the ischemic brain of mice. After microarray scanning and normalization, 1027 circRNAs were found to be differentially expressed in ischemic brains (fold change in expression ≥2; P<0.05), among which 914 were upregulated and 113 were downregulated. A hierarchical clustering of circRNA expressions is shown Figure 1a. The volcano and scatter plots show the significant variation of circRNA expression between the ischemic and sham brains (Figure 1b, 1c). The distribution of differentially expressed circRNAs in chromosomes is shown in Figure 2a. There were 27 intergenic, 30 antisense, 61 intergenic, 649 exonic, and 147 sense overlapping circRNAs among the upregulated circRNAs (Figure 2b). There were 5 intergenic, 85 exonic, and 23 sense overlapping circRNAs among the downregulated ones (Figure 2b). These results indicate that most of the differentially expressed circRNAs are transcribed from protein coding exons, and that only a few circRNAs are from other sources. These data suggest that the expressions of circRNAs in the ischemic brains differ from those in the sham group.

### Validation of the circRNA candidates using qRT-PCR

As more than 1000 circRNAs were found to have significant changes after stroke in the microarray assays, whether the results are reliable or not is not known. To confirm their reliability, 4 circRNAs with relatively high fold change from the microarray analysis were randomly selected for validation with qRT-PCR, including 3 up-regulated circRNAs (mmu_circRNA_40001, mmu_circRNA_013120, and mmu_circRNA_25329) and 1 down-regulated circRNA (mmu_circRNA_40806). Consistent with the results of the microarray analysis, the qRT-PCR data show that the circRNAs mmu_circRNA_40001 and mmu_circRNA_013120 were significantly upregulated, while the circRNA mmu_circRNA_40806 was significantly downregulated in the ischemic group (Table 1). However, the difference in mmu_circRNA_25329 expression was not statistically significant by qRT-PCR analysis.

**Table 1:** Quantitative real-time PCR validation for the expression of four circRNAs

CircRNAs	Microarray		PCR	
	Fold change	P-value	Fold change	P-value
mmu_circRNA_40001	11.84332	0.00978343	3.39668521	0.000512
mmu_circRNA_013120	11.2536866	0.00775987	2.427758835	0.001941
mmu_circRNA_25329	9.3216986	0.00971804	1.354407469	0.235249
mmu_circRNA_40806	0.290148753	0.00345225	0.417897758	0.000152

The expression levels of 4 circRNAs were determined by qRT-PCR. Each qRT-PCR assay was performed at least three times. *P < 0.05.

### MiRNA prediction of the confirmed circRNAs

To analyze the functions of the circRNAs, we identified and ranked the miRNAs targeted by the three confirmed circRNAs (mmu_circRNA_40001, mmu_circRNA_013120, and mmu_circRNA_40806) based on the mirSVR scores. The five highest-ranking miRNA candidates that are binding targets of each circRNA were identified as: 1) For mmu_circRNA_40001:mmu-miR-466f, mmu-miR-466i-5p, mmu-miR-669n, mmu-miR-1187, and mmu-miR-466c-5p; 2) For mmu_circRNA_013120: mmu-miR-6541, mmu-miR-669c-3p, mmu-miR-466f-5p, mmu-miR-669m-5p, and mmu-miR-466j; and 3) For mmu_circRNA_40806: mmu-miR-7038-3p, mmu-miR-20a-3p, mmu-miR-145a-3p, mmu-miR-346-3p, and mmu-miR-149-5p. We further analyzed the potential functions of these 15 miRNA targets of the three circRNAs.

### Prediction of putative target genes, their cell signaling pathways, and GO analyses

**Figure 3:**
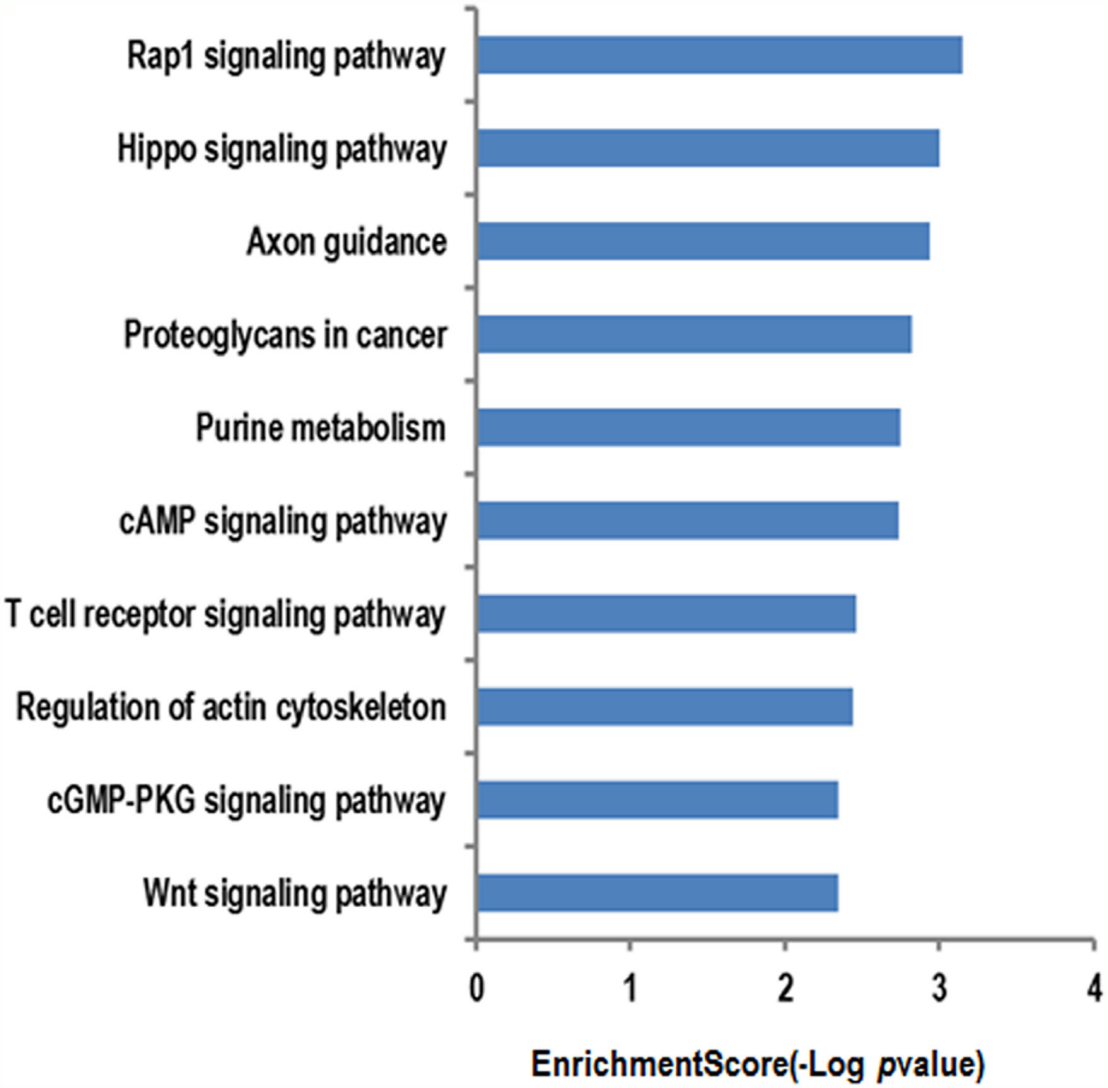
The KEGG database analyses of the cell signaling pathway annotations for target genes mediated by the circRNAs targeting miRNAs The vertical axis represents the miRNA targeting pathways associated with the circRNAs, and the horizontal axis represents the –LgP pathway values. LgP was the logarithm of p-value, and p < 0.05 was considered significant.

**Figure 4:**
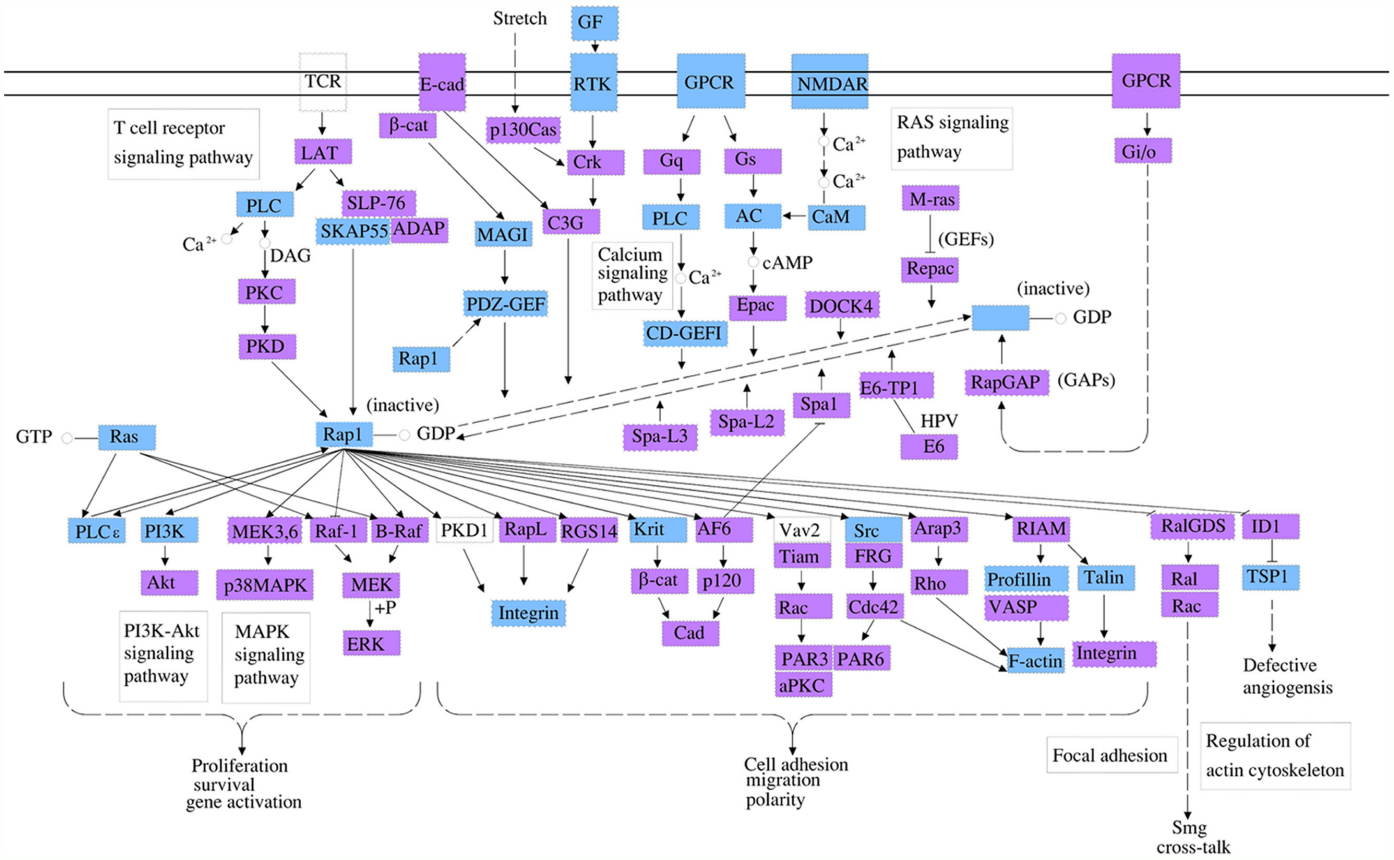
The Rap1 cell signaling pathway is a top pathway affected by the three identified circRNA associated miRNAs In this diagram, the genes that could be regulated by mmu_circRNA_40001, mmu_circRNA_013120, and mmu_circRNA_40806 are labelled in blue. Rap1 is a small GTPase that regulates a variety of biological processes, including cell adhesion, cell-cell junction formation, cell polarity, and cell death and survival. As a G protein, Rap1 changes between an inactive GDP-bound and an active GTP-bound conformation, which is controlled by diverse extracellular signals through the regulation of several unique guanine nucleotide exchange factors (GEFs) and GTPase activating proteins (GAPs). In addition to its roles in regulating cell-cell and cell-matrix interactions by regulating the function of integrins and other adhesion molecules in various cell types, Rap1 also controls the activities of Akt and MAP kinase (MAPK).

**Figure 5:**
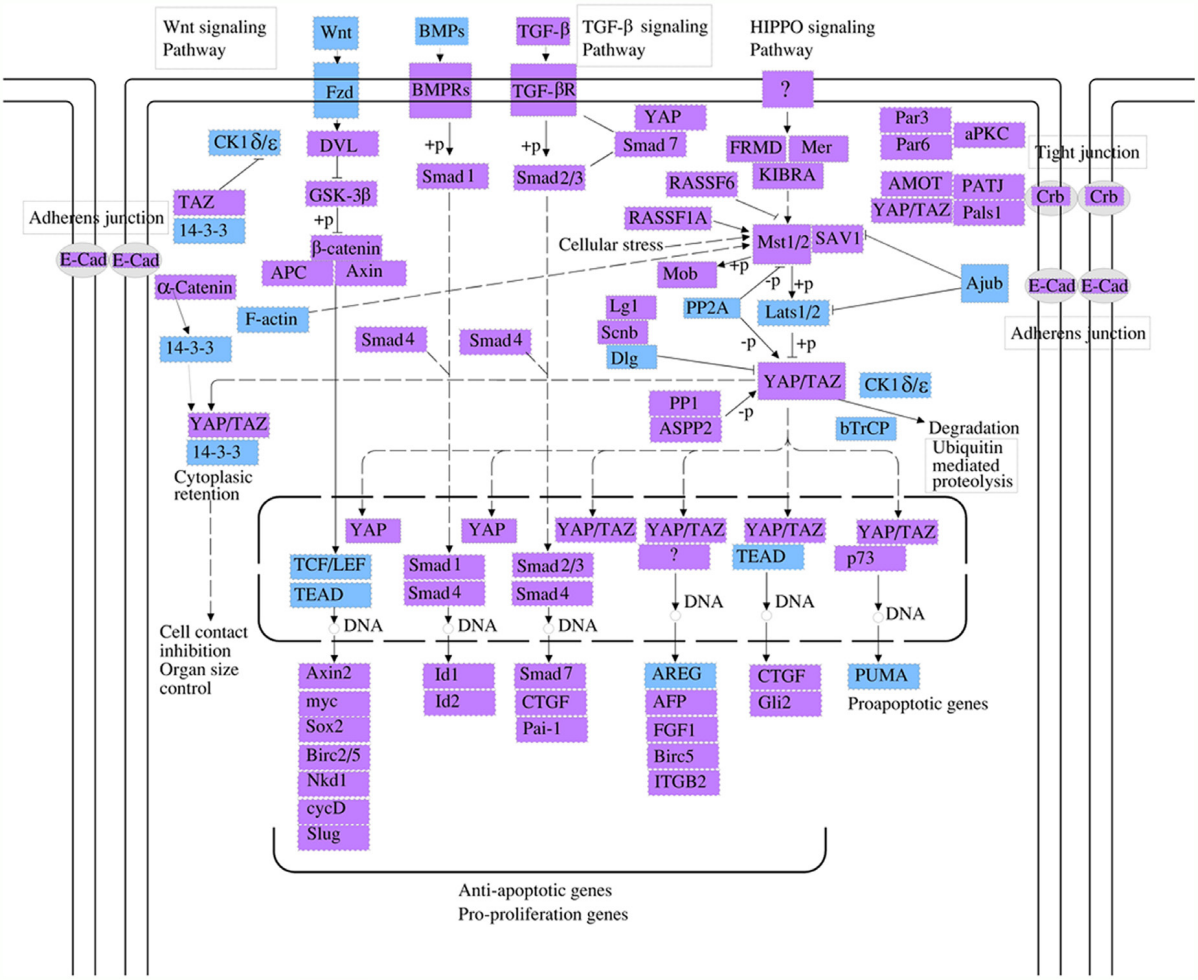
The hippo cell signaling pathway is another top pathway affected by the circRNAs identified in this study The genes regulated by mmu_circRNA_40001, mmu_circRNA_013120, and mmu_circRNA_40806 are identified in blue. The Hippo signaling pathway, named after the protein kinase Hippo, controls organ size in animals through the regulation of cell proliferation and apoptosis, and was originally identified in the fruit fly (Drosophila melanogaster). The mammalian counterpart of the hippo protein is MST1/2, which phosphorylates MOBKL1A/B, thus regulating cycle progression, growth, development, survival, and death.

For the further investigation of the functional roles of the 15 circRNA targeting miRNAs mentioned above, the putative target genes were predicted using the miRBase, miRanda, and TargetScan databases. The genes that were predicted by at least two of the three databases were used for further analysis. We used the KEGG database to analyze the cell signaling pathways of the predicted target genes; the associated 10 pathways are shown in Figure 3. The top two pathways were the Rap1 signaling pathway and the Hippo signaling pathway (Figure 3). The Rap1 pathway regulates cell adhesion, junction formation, proliferation, and survival [14], while the Hippo pathway is a major regulator of cell proliferation and survival in metazoans [15]. The genes regulated by the miRNAs are identified on the diagrams (Figures 4-5).

**Figure 6:**
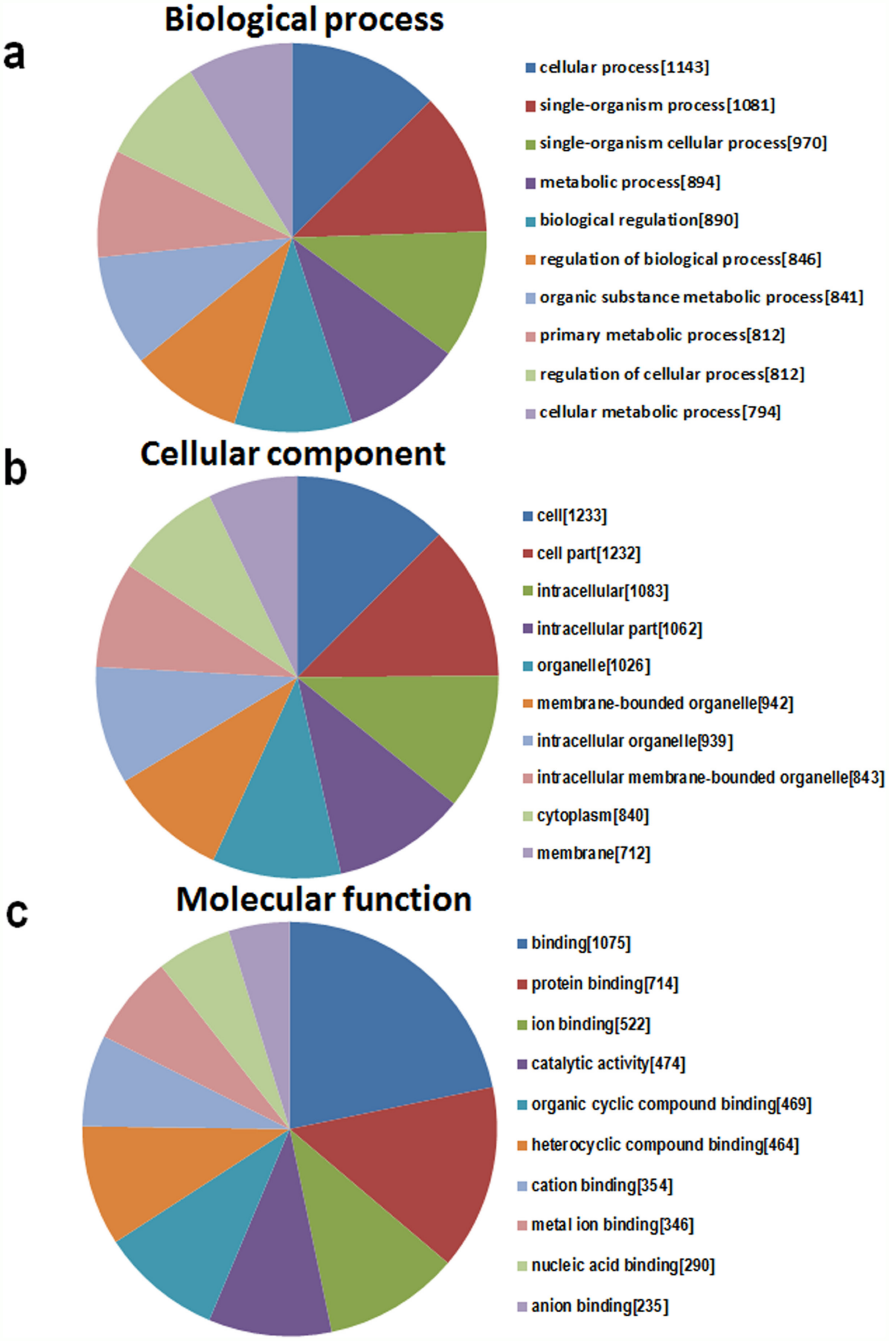
The GO annotations for target genes mediated by the miRNAs potentially regulated by mmu_circRNA_40001, mmu_circRNA_013120, and mmu_circRNA_40806 The GO analysis categorized the mRNAs into different groups under the theme of biological process (BP, **a**), cellular component (CC, **b**), and molecular function (MF, **c**). The gene numbers of each GO category are listed in bracket.

A major aim of GO is to analyze the functional enrichment of gene sets. According to the routine GO classification algorithms, we used it to characterize the three domains of the target genes functions, and the top 10 GO processes in each domain are presented in Figure 6. The three domains are 1) biological processes (Figure 6a), which describes a series of events accomplished by one or more organized assemblies of molecular functions, including the cellular process, the single organism process, and the single-organism cellular process; 2) cellular components, which describe the component of a cell, such as the membrane or the cytoplasm (Figure 6b); and 3) molecular functions, which describe activities that occur at the molecular level (Figure 6c).

### Prediction of interactions among circRNAs, miRNAs, and target genes

**Figure 7:**
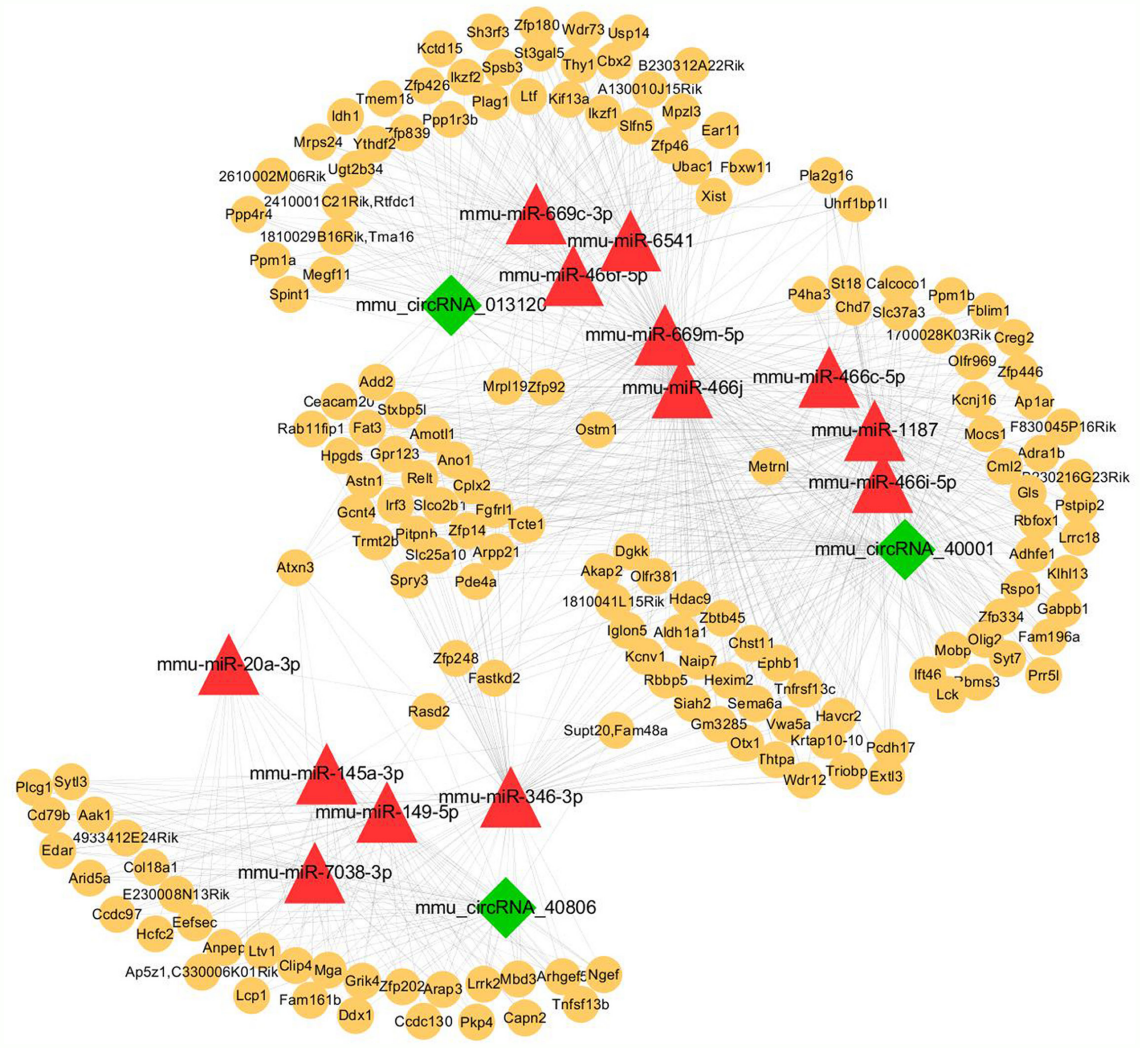
The circRNA-miRNA-gene network analysis The network consists of the 3 circRNAs, mmu_circRNA_40001, mmu_circRNA_013120, and mmu_circRNA_40806; (green nodes), miRNAs (red) and their target genes (yellow).

We then predicted the potential interactions between circRNAs and their target miRNAs using the Arraystar software for miRNA targets, according to the TargetScan and miRanda databases. An entire network of circRNA-miRNA-target gene interactions was delineated using the Cytoscape software (Figure 7). The results indicate that the potential miRNA targets include mmu-miR-466j, mmu-miR-669m-5p, mmu-miR-466f-5p, mmu-miR-1187, mmu-miR-466f, mmu-miR-466i-5p, mmu-miR-669n, and mmu-miR-466c-5p, exhibiting a larger interaction network. Because circRNA can serve as competing endogenous RNA for miRNA, circRNA may increase the expression of the target genes by removing the inhibitory effect induced by miRNA, according to the circRNA-miRNA-target gene network.

## DISCUSSION

In the present study, we provide novel results that suggest that circRNAs play important roles in cerebral injury after stroke, which is supported by a several pieces of evidence. First, we identified a total of 1027 circRNAs, including 914 upregulated and 113 downregulated circRNAs, and by using qRT-PCR, we confirmed changes in the expressions of the 3 selected circRNAs, mmu_circRNA_40001, mmu_circRNA_013120, and mmu_circRNA_40806. Second, the target genes of these confirmed circRNAs are predicted to be associated with many cell signaling pathways, including the Rap1 signaling pathway and the Hippo signaling pathway, which are important for cell survival, death, differentiation, proliferation, and neuroinflammation. Third, our further analyses suggest that the target genes of these circRNAs may modulate diverse biological processes, cellular components, and molecular functions. Fourth, the constructed network suggests a close relationship between the circRNAs, miRNAs, and their target genes. Taken together, our results provide strong evidence that circRNAs are extensively involved in brain injury after stroke.

Regulatory RNAs, such as miRNAs (miRNAs) or long non-coding RNAs (lncRNAs), have been implicated in many biological processes and human diseases, such as cancers [16–18]. With the rapid advances in high-throughput sequencing and bioinformatic analysis, recent studies have shed light on a new class of RNA, circRNAs, which are endogenously expressed as single-stranded, covalently closed circular molecules [19]. Recent evidence has also shown that circRNA can serve as competing endogenous RNAs (ceRNAs) for miRNAs [20]. A circRNA may contain multiple miRNA binding sites and have adsorption effects on miRNAs. Therefore, a circRNA can remove the inhibitory effect of an miRNA on its target genes, thus increasing the expression of the target genes. For example, ciRS-7 contains miRNA-7 binding sites, thereby suppressing miR-7 activity, resulting in increased levels of miR-7 targets [21]. In addition, using bioinformatic analysis Lin, *et al.* find that oxygen-glucose deprivation/reoxygenation (OGD/R) alteres the expression of circular RNA, and that the upregulated expression of mmu-circRNA-015947 interacts with miRNAs (mmu-miR-188-3p, mmu-miR-329-5p, mmu-miR-3057-3p, mmu-miR-5098, and mmu-miR-683) [22]. Nevertheless, there have been few *in vivo* reports on the profile and function of specific circRNAs in stroke.

In the present study, our circRNA expression profiles revealed that 914 circRNAs were aberrantly upregulated and 113 circRNAs were downregulated in ischemic brains compared to those from the sham group. These results suggest that the identified circRNAs that were significantly differentially expressed might be implicated in stroke-induced brain injury, as we have confirmed with qRT-PCR that the three circRNAs, mmu_circRNA_40001, mmu_circRNA_013120, and mmu_circRNA_40806, were significantly changed in ischemic brains compared with those from the sham group. Therefore, our findings indicated that these circRNAs represent potentially valuable diagnostic biomarkers for stroke.

To analyze the functions of the three circRNAs, we first predicted the circRNA/miRNA interactions based on conserved seed sequence matches. We ranked miRNA candidates that were binding targets of each circRNAs based on the mirSVR and remained the five highest-ranking miRNA candidates for each circRNA. In order to further explore the functions of circRNAs-miRNAs axes, we then predicted the target genes of these related miRNAs. Then, we used KEGG pathway analysis to functionally annotate the predicted target genes of these miRNAs. According to our annotation, the top signaling pathways affected by the circRNAs-miRNAs axes were the Rap1 signaling pathway, the Hippo signaling pathway, the T-cell receptor signaling pathway, and the Wnt signaling pathway. Previous studies have found that Rap1 may induce hepatic ischemic reperfusion injury through the promotion of the inflammatory neutrophil response [23] and that the Hippo signaling pathway plays an important role in cell growth, proliferation, apoptosis, and dendritic remolding [24]. In addition, the transcription cofactor Yes-associated protein (YAP) in the Hippo pathway has been suggested to elicit both beneficial and detrimental effects on regeneration and fibrogenesis after acute ischemic kidney injury [25]. Furthermore, the Wnt signaling pathway has been found to regulate functional recovery after stroke [26–28]. Therefore, circRNAs seem to be integrally involved in brain injury after stroke. Meanwhile, we also illustrated the GO terms, which included the biological process, cellular component and molecular function of the target genes of these miRNAs. These functional analysis results further suggest that these circRNAs are important for controlling diverse biological processes, cellular signaling pathways, and protein activities in ischemic brains by regulating miRNAs and their tatget genes.

To providing visual information for the “miRNA sponge” function of circRNAs, we constructed a circRNA-miRNA-target gene interaction network with Arraystar's homemade miRNA target prediction software based on TargetScan & miRanda. These networks provided an important reference value for studying the interaction of the differentially expressed circRNAs, miRNAs and their potential targets. Although the verified circRNA-miRNA- target gene axis has not been previously reported, several molecules in the axis are involved in certain pathophysiological processes. For example, among the observed circRNA/miRNA interactions, the potential miRNA targets of mmu_circRNA_40806 include miR-149-5p, mmu-miR-346-3p, and mmu-miR-20a-3p. A previous study indicated that miR-149-5p regulates the expression of the pro-apoptotic Bcl-2 proteins DP5 and PUMA, which induce human β-cell apoptosis [29]. In addition, another study found that miR-149-5p, as a tumor suppressor, is associated with cellular migration, proliferation, and apoptosis in renal cell carcinoma [30]. Furthermore, another previous study indicates that mmu-miR-346-3p regulates cell viability through the mTOR signaling pathway in mouse embryonic fibroblast cells treated with polyethylenimine [31]. Since mmu_circRNA_40806 is a potential sponge for mmu-miR-20a-3p, we therefore speculate that mmu_circRNA_40806 might competitively bind with mmu-miR-20a-3p and relieve the inhibitory effects on the associated target genes including Hcfc2, Aak1, Capn2, and Tnfsf13b in the network (Figure 7). MiR-20a-3p has been found to be involved in the pathogenesis of cancers, such as gastric and breast cancers [32, 33]. Because mmu-circRNA is a predicted sponge of these miRNAs, it may compete with them, thereby inhibiting target gene expression and participating in the pathogenesis of cerebral ischemia/reperfusion. Thus, further investigation of these novel circRNAs as miRNA sponges is therefore worthwhile, which may help us understand the mechanisms underlying brain injuries after stroke.

However, there are some limitations in this current study. First, although we have identified more than 1000 circRNAs that were significantly changed after stroke, none of their functions have been identified. Second, a time course study has not been conducted to clarify the possible dynamic changes in circRNA expression in the ischemic brain after stroke. Third, the relationship between circRNAs and miRNAs has not been studied. Here, we only analyzed three circRNAs and their five highest-ranking miRNA candidates, respectively. In fact, the roles of miRNAs in cerebral ischemia have been extensively studied in the past few years. For example, Uhlmann *etal* foud that miR-1264/1298/448 cluster peaked in the circulation around 3 hours after reperfusion and gradually decreased thereafter, suggesting a potential to serve as biomarkers for reperfusion in the acute phase [34]. Another study indicated that mR-210 was a crucial ischemic stroke-associated miRNAs and a potential target for the stroke therapy [35]. We have investigated the miRNAs profiles in the brain following focal cerebral ischemia in mice and identified 118 significantly expressed miRNAs [36]. Thus, further analysis of the relationship between the identified circRNAs in the present study and their interaction with miRNAs, which have been previously reported to be associated with brain ischemia, may expand our understanding in the previous mechanism of miRNAs in cerebral ischemia. In the future, a tome course study for selected circRNAs will be conducted to show their dynamic changes after stroke in the ischemic brain. In addition, functional experiments should be performed to demonstrate a mechanistic role for some of the differentially regulated circRNAs. Furthermore, how circRNA affects miRNA will be studied. At last, gene manipulation of circRNAs with knockdown or over expression will be conducted *in vivo* to study the functions of circRNAs.

In conclusion, the identified reservoir of circRNAs provides preliminary data for searching for candidate for stroke diagnosis, and the predicted circRNA-miRNA-target gene network may provide potential insights in the elucidation of the mechanisms of brain injury for stroke.

## MATERIALS AND METHODS

### Focal cerebral ischemia in mice

All procedures in this study were conducted according to the guidelines set by the University Animal Care and Use Committee of Capital Medical University.

Adult male *C57BL/6* mice weighing 20-22g were purchased from Vital River Laboratory Animal Technology Co. Ltd. (Beijing, China) and were housed in a temperature controlled room with a 12 h light/dark cycle. The animals received free access to food and water.

Transient focal cerebral ischemia was induced by right middle cerebral artery occlusion for 45 min, as previously described [37, 38].Briefly, anesthesia was induced by inhalation of 5% isoflurane (Lunan Pharmaceutical Group Corporation; Shandong, China) in a 30% O_2_ and 68.5% N_2_O mixture, maintained with 2% isoflurane inhalation. Rectal temperature was maintained at 37 ±0.5 °C with a feedback heating pad during surgical procedures. Sham-operated animals underwent anesthesia and surgery without MCA occlusion. All mice were placed in a post-operative cage, and kept warm and undisturbed for a minimum of 2 h for observation. The mouse brains were removed 48 h after reperfusion, the infarct regions were collected as previously reported [39], and quickly frozen in a liquid nitrogen can.

### CircRNA microarray and analysis

Total RNA was isolated from the brain tissues using Trizol reagent (Invitrogen, Carlsbad, USA) according to the manufacturer’s instructions. The RNA sample concentrations were determined by OD260/OD280 using a NanoDrop ND-1000 instrument. The integrity of the RNA was assessed by electrophoresis on a denaturing agarose gel.

The sample preparations and microarray hybridizations were performed according to Arraystar’s standard protocols. Briefly, the total RNA was digested with Rnase R (Epicentre, Inc.) to remove linear RNAs. The enriched circular RNAs were then amplified and transcribed into fluorescent cRNAs, utilizing a random priming method. The labeled cRNAs were hybridized onto the Arraystar Mouse circRNA Array (8x15K, Arraystar) and the hybridized arrays were washed and scanned with the Agilent Scanner G2505C. The Agilent Feature Extraction software (version 11.0.1.1, USA) was used to analyze the acquired array images. Quantile normalization and subsequent data processing were performed using the R software package. CircRNAs that were differentially expressed between the ischemic and control groups were conveniently estimated by fold-change filtering and the Student's t-test. False discovery rate (FDR, <0.05) was calculated in order to correct the P-value. CircRNAs exhibiting fold changes≥ 2.0 and p-values < 0.05 were considered to be significant.

### Quantitative reverse transcription real-time polymerase chain reaction (qRT-PCR)

The expressions of 4 randomly selected circRNAs were validated with qRT-PCR. Total RNA was extracted from the brain tissues using Trizol Reagent (Invitrogen). SuperScriptTM III Reverse Transcriptase (Invitrogen) was used to synthesize the cDNA according to the manufacturer’s instructions. QRT-PCR was performed in the ViiA 7 Real-time PCR System (Applied Biosystems), which was performed in a 10 μl reaction volume, including 2 μl of cDNA, 5 μl 2× Master Mix, 0.5 μl of Forward Primer, 0.5 μl of Reverse Primer, and 2 μl of double distilled water. The PCR conditions were 95 °C denaturation for 10 min, 95 °C for 10 s, and 60 °C for 60 s, repeated for 40 cycles. All samples were normalized to the signals generated from the GAPDH housekeeping gene. The primers used for the qRT-PCR analysis are shown in Table 2. The relative expressions of circRNAs were calculated using the formula 2^-(Δ*C*t ischemia - Δ*C*t control)^ [40].

**Table 2:** Primers used for RT-qPCR analysis

Primer name	Primer sequence
mmu_circRNA_40001	F:5’ ATGGGAGGAGATCGCAACA3’ R:5’ CAGTTCCCACCAGCCATTT 3’
mmu_circRNA_013120	F:5’ GTGAGTCTGGGAGGATGGAT3’ R:5’ CCAATCTGGCACACACACTT3’
mmu_circRNA_25329	F:5’ GGTGGATATGTCTGGGTTGA3’ R:5’ GACTCTTTGCTTCGCCG3’
mmu_circRNA_40806	F:5’ CGGTATTGGAATGAGTGGAA3’ R:5’ TATGAGGGAGGACTCTTAGCA3’
GAPDH	F:5’ CACTGAGCAAGAGAGGCCCTAT3’ R:5’ GCAGCGAACTTTATTGATGGTATT3’

### MiRNA targeting gene prediction and computational analysis

In order to further investigate the functional roles of miRNA, the putative targets of the miRNAs were predicted with the miRBase, miRanda and TargetScan programs. Gene Ontology (GO) analysis (http://www.geneontology.org) was used to illuminate the biological process, cellular component and molecular function of the mRNAs. The pathway analysis (KEGG database) was also carried out for mapping genes to KEGG pathways. The p-value denotes the significance of GO and pathway terms (p-value < 0.05). The false discovery rate (FDR) was used to judge p-values.

### CircRNA-miRNA co-expression network

CircRNA and miRNA interactions were predicted with the Arraystar's homemade miRNA target prediction software based on TargetScan and miRanda [41, 42]. To visualize their interactions, a total of 3 circRNAs, whose expressions were confirmed, and 13 miRNAs were selected to generate a circRNA-miRNA-gene network, using the Cytoscape software.

### Statistical analysis

Statistical analysis was conducted using the Student’s t-test. All data are presented as mean ± SE, and the significance was set at p<0.05.
